# Patients with relapsed/refractory hairy‐cell leukemia

**DOI:** 10.1002/cnr2.1495

**Published:** 2021-07-12

**Authors:** Jérôme Paillassa, Xavier Troussard

**Affiliations:** ^1^ Department of Hematology Academic Hospital of Angers Angers France; ^2^ Laboratory of Hematology Academic Hospital of Caen Caen France

**Keywords:** HCL, OS, PNA, relapses, RFS

## Abstract

**Background:**

Hairy cell leukemia (HCL) is a rare chronic B‐cell neoplasm with good long‐term prognosis. First and second‐line therapies include purine nucleoside analogues (PNAs) and rituximab, but until recently, limited alternative options were available for patients with two or more relapses.

**Aim:**

The aim of this study is to describe our real‐life experience with HCL patients in third and fourth‐line therapies.

**Methods and Results:**

Data from 49 HCL patients with two or more relapses, including 16 patients with three or more relapses, were collected from the French retrospective HCL cohort covering the period from 1980 until 2011. They were analyzed to assess hematological response, relapse free survival (RFS) and overall survival (OS) after third (L3) and fourth line (L4). The median age at diagnosis was 53 years. PNAs were the most frequently used treatments. As L3 therapy, 29 patients received PNAs (66%) and 15 (34%) other treatments (rituximab [11%] or interferon [7%] alone or in combination [16%]). The distribution of L4 treatments was similar. The overall hematological response rate (OHRR) after L3 was 97% (complete hematological response 86%) with a 40% five‐year cumulative incidence of relapse (CIR), a median RFS of 104 months, and a median OS of 235 months. After L4, the OHRR was 94% with a two‐year CIR of fourth relapse of 27%. Eleven secondary cancers (5‐year cumulative incidence of 12%) were diagnosed in 10 patients. Patients with ≥2 relapses experience frequent further relapses, with increasingly shorter time to next treatment as the number of treatment lines increases. Furthermore, treatment strategies are associated with substantial toxicities.

**Conclusion:**

All these points lead to the need for novel treatments.

## 1 INTRODUCTION

Hairy cell leukemia (HCL) is a rare, chronic B‐cell neoplasm recognized as a distinct entity of leukemia by the World Health Organization (WHO) in 2001.^1^ It is characterized by abnormal, clonal, mature B‐cells with hairy projections found in blood, bone marrow, spleen or liver.^2^, ^3^ Patients usually present with infections, splenomegaly or pancytopenia and, in some cases, with unusual autoimmune and/or bone manifestations mimicking multiple myeloma.^4^ Asymptomatic HCL patients can be diagnosed by discovery of hairy cells during routine peripheral blood analyses. Hairy cells express CD11c, CD25, CD103, and CD123. The BRAF V600E mutation of the B‐raf proto‐oncogene is considered as the HCL‐defining mutation^5^ suggesting possible new diagnostic and treatment approaches. HCL represents 2% of all leukemia with a worldwide normalized incidence ratio of 0.5 per 100 000 person‐years for men and 0.1 per 100 000 person‐years for women and a median age at diagnosis of 63 years in men and 59 years in women in France.^6^ If splenectomy was the historical treatment for HCL,^7^ the first effective systemic treatments appeared in the mid‐eighties with the introduction of interferon‐α_1_ and α_2_ and of purine nucleoside analogues (PNAs), namely pentostatin (2′‐deoxycoformycin)^8^ and cladribine (2′‐chlorodeoxyadenosine).^9^ Interferon‐α induces mostly only partial responses albeit with high overall response rates (ORRs),^10^ but PNAs really transformed the treatment of HCL with ORRs higher than 85%, complete hematologic responses (CHRs) for most patients and prolonged relapse free survival (RFS) with median values up to 15 years.^9^, ^11^, ^12^, ^13^, ^14^ PNAs are the established first‐line option for symptomatic HCL patients and remain the first‐choice treatment, with or without rituximab, for patients who relapse.

For patients relapsing more than twice, using the same PNA or switching for another PNA is the frequently chosen option. However, this practice presents several limitations. First, response rates and duration of response (DOR) decrease at each relapse. In the study published by Zinzani and colleagues, the median DOR was 2.2 years after the third line treatment, then 1.6 years after the fourth one.^15^ In the same way, in the study of Else et al., CR rate and RFS decrease at each treatment line: 81%, 66%, 50%, and 16, 11, 6.5 years, after first, second and third line, respectively.^16^ Moreover, there is a cumulative toxicity associated with the use of several lines of PNA, like infections and second cancers. For these relapse/refractory patients, additional options are now available including recombinant immunoconjugates targeting CD22, BRAF inhibitors and BCR inhibitors.^17^

In this article, we present the long‐term follow‐up results from a subset of the French retrospective HCL cohort for patients with two or more relapses, focusing on overall survival (OS), RFS for patients with three (L3) and four (L4) treatment lines before the appearance of the recent treatments.

## 2 MATERIAL AND METHODS

### 2.1 Design

The initial French retrospective HCL cohort included 487 patients from 36 centers in France diagnosed with HCL between January 30, 1980 and September 20, 2011, with an end of follow‐up in December 2012.^18^ In January 2018, it was decided to update the database to get follow‐up data until June 2018.

### 2.2 Study centers

A list of 60 French centers known to treat patients with HCL was provided by the French Innovative Leukemia Organization. These centers were sent an e‐mail that offered them to participate in the initial survey. Thirty‐six of them agreed to take part. When it was decided to update the database, they were contacted again, inviting them to provide follow‐up data for their patients. Nineteen centers answered positively.

### 2.3 Study population

For the initial survey, investigators were to include all consecutive patients aged 18 or more and diagnosed with HCL according to the 2008 and 2016 WHO classifications (including morphological, flow cytometric analysis of circulating blood, bone marrow or tissue specimen), assuming that they signed a written informed consent form. We collected follow‐up data until June 2018 for 279 patients. In this paper, we present the results for the 49 with two or more relapses (L3: patients requiring a third line of treatment) and 16 L4 patients (L4: patients requiring a fourth line of treatment); these patients have been treated by 12 centers. Treatment criteria used by all centers were those defined by the *Société Française d'Hématologie* in 2014: symptomatic splenomegaly, recurrent or severe infections, cytopenia (involving at least one cell type: hemoglobin <10 g/dl, platelets <100 × 10^9^/L, neutrophils <1 × 10^9^/L).^19^

### 2.4 Data collection

For each patient, the investigators collected the following information retrospectively from the patients' record: patient's characteristics at baseline including demographics, date of diagnosis, clinical and biological presentation (hemoglobin, platelet count, white blood cell count, neutrophil count, flow cytometry analysis); as well as follow‐up data by treatment line with start and end dates for each treatment, response to treatment (date, complete hematological response [CHR], partial hematological response [PHR], failure), date of relapses, second solid cancer (date, histology), second hematological malignancies (date, WHO 2016 classification^20^), date and cause of death and date of last news. Microsoft Excel® and Filemaker® were used for data collection.

### 2.5 Statistical methods

Patients' baseline characteristics are described for the following subgroups: L1–L2 (first line and second line of treatment), L3 and L4. Survival curves for OS, RFS and time to next treatment (TTNT) were determined using the non‐parametric Kaplan–Meier method. OS was defined as the time between start of L3 or L4 and the date of death or last news (censoring date). RFS was defined as the time between the date of start of the treatment line (L3 or L4) and the date of relapse or death, event‐free patients being censored at the date of last news. TTNT was defined as the time laps between start of L3 or L4 and the start of next treatment line or death, event‐free patients being censored at the date of last news. Cumulative incidences of relapse (CIR) were determined along with its confidence intervals (95% CI) considering death as a competing risk. Hematological response to treatment was assessed by the investigators and hematological response rates after L3 and L4 are provided. CHR was defined as the disappearance of organomegaly and normalization of the hemogram (a bone marrow evaluation was not mandatory). PHR was defined as the reduction of organomegaly by at least 50%, the disappearance of cytopenias, and <5% circulating hairy cells. Overall hematological response rate (OHRR) was defined as the sum of CHR and PHR rates. SPSS® (version 16.0) and R® (version 3.5.3) softwares were used for the statistical analyses.

## 3 RESULTS

We analyzed the data from 49 patients with two or more relapses (18% of the updated cohort of 279 patients) from 12 French centers. Of these, 16 (6% of the cohort) had three or more relapses. They were all diagnosed with HCL between 1980 and 2011 and their median duration of follow‐up after the date of second relapse was 67 months (range 2–373). Five patients with a second relapse received no L3 treatment. The median duration of follow‐up for L3 patients was 94 months (range 0–373) after the start of L3. For the 16 L4 patients, it was 50 months (range 7–343) after the start of L4.

### 3.1 Patients' characteristics at baseline

At diagnosis, patients with two or more relapses were 6 years younger than patients from the original cohort of 279 patients (median 53 vs. 59 years) and had lower hemoglobin (112 vs. 120 g/L, p = .011) and platelet count (77.5 vs. 93.5 × 10^9^/L, p = .038), slightly lower white blood cell (2.3 vs. 2.7 × 10^9^/L, p = .613) and neutrophil counts (0.8 vs. 1.0 × 10^9^/L, p = .056). The two sets of patients were similar in terms of rate of infectious disease at diagnosis (18% vs. 21%) (Table 1).

**TABLE 1:** Patients' characteristics at baseline

Age at HCL diagnosis (years), median [range]	53 [29–80]
Hemoglobin (g/dl), median [range]	11.2 [5.3–15.8]
Platelet count (× 10^9^/L), median [range]	77.5 [7.4–157]
White blood cell count (× 109/L), median [range]	2.3 [0.5–63.5]
Neutrophil count (× 10^9^/L), median [range]	0.79 [0.1–9.1]
Infectious disease at diagnosis, *n* (%)	9 [18]

### 3.2 During the 1980–2011 period, PNAs were the most frequently used treatment in the third and fourth lines setting

Fifty‐one percent of patients received PNAs in first line, 69% in second line. Forty‐five percent of patients received interferon α in first line, 20% in second line. Three patients underwent splenectomy as L1 treatment, one as L2 treatment (Table 2).

**TABLE 2:** Treatment of patients in first, second, third and fourth line (L1, L2, L3, L4)

Treatment	1st line (*N* = 49)	2nd line (*N* = 49)	3rd line (*N* = 44)	4th line (*N* = 16)
Purine nucleoside analogues	25 (51%)	34 (69%)	29 (66%)	9 (56%)
Cladribine	20 (41%)	22 (45%)	12 (27%)	6 (38%)
Pentostatin	5 (10%)	12 (24%)	17 (39%)	3 (19%)
Others			15 (34%)	7(44%)
Splenectomy	3 (6%)	1 (2%)		
Rituximab	‐	5 (10%)	5 (11%)	2 (13%)
Interferon α	22 (45%)	10 (20%)	3 (7%)	3 (19%)
Rituximab + cladribine			4 (9%)	‐
Rituximab + Pentostatin			1 (2%)	1 (6%)
Cladribine + interferon α			1 (2%)	‐
Rituximab + interferon α			‐	1 (6%)
Rituximab + bendamustine then vemurafenib			1 (2%)	‐
Untreated			5 (10%)	‐

*Note*: Results are presented as *N* (%).

After their second relapse, 5 of 49 patients (10%) remained untreated. As L3 therapy, 29 of 44 patients (66%) received a single agent PNA, either cladribine (*N* = 12/44, 27%) or pentostatin (*N* = 17/44, 39%). A further five patients (*N* = 5/44, 11%) received cladribine in combination with rituximab (*N* = 4/44, 9%) or with interferon α (*N* = 1/44, 2%) The other third‐line treatments (*N* = 15/44, 34%) included rituximab (*N* = 5/44, 11%), interferon α (*N* = 3/44, 7%), and a combination of rituximab and bendamustine followed by vemurafenib (*N* = 1/44, 2%) (Table 2).

All patients with a third relapse received a L4 treatment. Most patients received a single agent PNA (*N* = 9/16, 56%), twice as many cladribine (*N* = 6/16, 38%) than pentostatin (*N* = 3/16, 19%). Other treatments were given to 7 of 16 patients (44%) and included interferon α (*N* = 3/16, 19%), rituximab (*N* = 2/16, 13%) monotherapies, and rituximab combined with either pentostatin or interferon α for one patient each (6%).

### 3.3 Outcomes after L3

**FIGURE 1:**
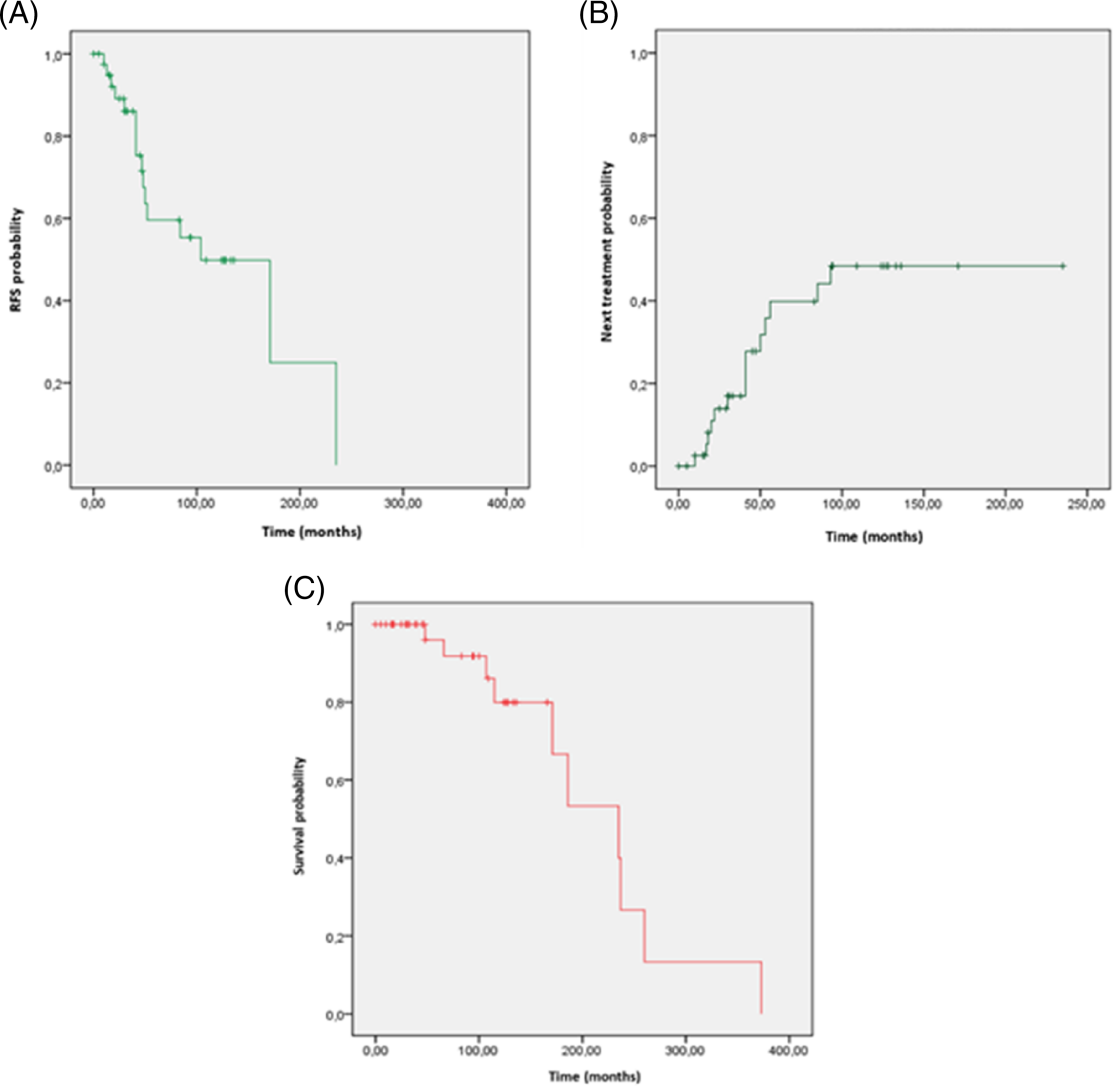
Relapse Free Survival (RFS), Time to Next Treatment (TTNT) and Overall Survival (OS) from start of L3. (A), RFS, (B), Time to Next Treatment, (C), OS from diagnosis

After excluding the five patients with second relapse who never received an L3 treatment, the five‐year CIR after L3 treatment was 40% (95% CI 22%–58%) with a median RFS of 104 months (95% CI 48–160 months), a mean TTNT of 143 months (95% CI 107–179 months, median unreached) and a median OS of 235 months, (95% CI 155–15) (Figure 1). After L3 treatment, 5‐year OS was 96% (95% CI 92.1%–99.9%) and 10‐year OS was 79.9% (95% CI 70.7%–89.1%). Disease progression (5/14, 36%) and secondary cancer (3/14, 21%) were the main causes of death. The OHRR after L3 was 43 of 44 (97%), with 38 of 44 patients (86%) achieving a CHR and 5 of 44 patients (11%) achieving a PHR (Table 3).

**TABLE 3:** Response to third‐ and fourth‐line treatments

	*N*	CHR^a^	PHR^a^	OHRR^a^	No response^a^
Third‐line treatment					
PNA	29	28 (97%)	—	28 (97%)	1 (3%)
Cladribine	12	12 (100%)	—	12 (100%)	—
Pentostatin	17	16 (94%)	—	16 (94%)	1 (6%)
Others	15	10 (67%)	5 (33%)	15 (100%)	—
Rituximab	5	3 (60%)	2 (40%)	5 (100%)	—
Interferon α	3	2	1	3	—
Other(s)	7	5 (71%)	2 (29%)	7 (100%)	—
Total	44	38 (86%)	5 (11%)	43 (98%)	1 (2%)
Fourth‐line treatment					
PNA	9	7 (78%)	2 (22%)	9 (100%)	—
Cladribine	6	6 (100%)	—	6 (100%)	—
Pentostatin	3	1 (33%)	2 (66%)	3 (100%)	—
Others	7	4 (57%)	2 (29%)	6 (86%)	1 (14%)
Rituximab	2	2	—	2	—
Interferon α	3	2	—	2	1
Other(s)	2	—	2	2	—
Total	16	11 (69%)	4 (25%)	15 (94%)	1 (6%)

Abbreviations: CHR, Complete hematologic response; OHHR, Overall hematological response rate; PHR, Partial hematological response.

^a^ *N* (%); note that percentages are not calculated when the number of patients is <5.

### 3.4 Outcomes after L4

For the 16 patients in third relapse, the median RFS was 36 months (95% CI 20–52 months), with a mean TTNT of 98 months (95% CI 16–181 months, median 36 months [95% CI 16–56 months]) and a median OS of 93 months (95% CI 43–143 months). After L4 treatment, five‐year OS was 62.3% (95% CI 47.3%–77.3%) and 10‐year OS was 41.6% (95% CI 26%–57.2%). The OHRR after L4 treatment was 15 of 16 (94%) with 11 of 16 CHR (69%) and 4 of 16 PHR (25%) (Table 3). After L4 treatment, nine patients relapsed (56%) with a 2‐year CIR of 27%.

### 3.5 Secondary cancers

Ten patients (10/44, 23%) experienced at least one secondary cancer, corresponding to an overall five‐year cumulative incidence of second cancers of 12% (95% CI 4–24%). A total of 11 secondary cancers were reported, seven solid cancers (four non‐melanoma skin cancers, one prostate cancer, one kidney cancer, and one esophagus cancer) and four hematological malignancies (two myelodysplastic syndromes, one monoclonal gammopathy of unknown significance and one diffuse large B cell lymphoma).

## 4 DISCUSSION

We analyzed the data of patients with two and more relapses in the updated 279 patients of the French retrospective HCL cohort.^21^ We also analyzed the data of patients with three and more relapses. The long‐term prognosis of these patients with multiple relapsing HCL is reasonably good, even if not as good as the prognosis of the average HCL patients from the whole cohort.

More than one third (36%) of L3 treated patients relapsed with a five‐year CIR of 40%, a median RFS of 104 months, an unreached median TTNT and a median OS of nearly 20 years. After L4 treatment, a higher proportion of patients relapsed (56%) with a two‐year CIR of 27% and shorter RFS (median 36 months), TTNT (median 36 months), and OS (median 93 months).

Response data from this study should be interpreted with caution. Indeed, more recent trials and other clinical data usually present response data based on peripheral blood counts supplemented by bone marrow status. There is even an interest to assess responses with negative minimal residual disease (MRD). Such data were not collected in the French registry and would be hard to retrieve retrospectively.

The OS observed in our patients is rather long, despite the fact that they had two or more relapses; however, OS is significantly decreased compared to the overall HCL patients. For the complete updated French retrospective cohort, the median OS was 27 years; the median OS for L3 patients was nearly 20 years and that for L4 patients, nearly 8 years. These observations are in line with other results published in the literature.^12^, ^13^, ^14^, ^15^, ^16^, ^22^, ^23^, ^24^, ^25^, ^26^, ^27^, ^28^, ^29^, ^30^, ^31^, ^32^, ^33^ Similarly, there is a progressive decrease of the RFS (L1: 136 months, L2: 86 months, L3: 104 months, L4: 36 months) and of the TTNT (L1: 172 months, L2: 131 months, L3: unreached, L4: 36 months) with increasing treatment lines. Decreasing RFS with treatment lines was reported by Dearden et al. (L1: 16 years, L2: 11 years, L3: 6.5 years).^34^

As reported in other series, the occurrence of second malignancies is common in patients with HCL. In the whole cohort, cancer was the first cause of death with a 10‐year cumulative incidence (considering death as a competing risk) of 15% for all cancers, 11% for solid cancers and 5% for hematological malignancies.^21^ In the subset of patients with two or more relapses, 10 patients experienced 11 cancers, seven solid cancers and four hematological malignancies, corresponding to a 5‐year cumulated incidence of 12%. Referring on the treatment used in our cohort, single‐agent PNA was the most frequently used treatment for first (75%) and second lines (69%). This was also the case after the second and third relapses with respectively 59% and 57% of the patients treated with PNA. In their study assessing long‐term outcomes of 121 HCL patients, Zinzani et al. reported 86%, 79%, and 77% of the patients treated with PNAs for second, third, and fourth line treatments.^15^

Our cohort consists of patients who were diagnosed with HCL between 1980 and 2011 and followed up until June 2018. Due to the retrospective nature of our study and to the long duration of follow‐up, our results cannot reflect the current approach of HCL therapy, which is clearly evolving. If PNAs remain the established L1 treatment for symptomatic HCL patients, chemoimmunotherapy combining PNAs and rituximab is now recommended in second‐line, while immunoconjugates targeting CD22, BRAF inhibitors and BCR inhibitors should be considered for relapsed or refractory patients.^17^

Our study suffers from several limitations: first it is a retrospective study. Second, it involves patients treated during the period of 1980 and 2011; standards of assessment and standards of treatment have evolved during that period of time with an inevitable impact on overall outcome.^17^ Our results must be weighted by the fact that they are not based on the current standard of treatment assessments; in particular bone marrow and MRD assessments were not available in our data.

The pathophysiology of HCL is now better understood, opening the way for new therapies including combined chemoimmunotherapy, immunotoxins targeting CD22 (moxetumomab pasudotox), BRAF inhibitors (vemurafenib, dabrafenib), MEK inhibitors (trametinib, cometinib), and BTK inhibitors (ibrutinib). Prospective studies on these new treatments use different assessment criteria and focus on marrow response and absence of MRD. In an open‐label, single‐arm study, 80 HCL patients with two or more prior systemic therapies were treated with moxetumomab pasudotox. Complete marrow response was achieved for 41% of the patients, with no MRD (assessed by immunohistochemistry) for 85% the complete responders (34% of all the patients). After a follow‐up of 17 months, the median for time to relapse remained unreached.^35^ Tiacci et al. report ORRs of 96% and 100% with 35% and 42% of CR and 27% relapses at one year in a phase two study involving two groups of 26 HCL patients refractory to PNAs treated with vemurafenib.^36^ The combination with rituximab and vemurafenib was tested in a small series: the authors reported a 100% ORR, a 96% CR rate, a 63% negative MRD rate, and a 15% relapse rate after a follow‐up of 26 months.^37^ In 28 patients with a median of four prior lines treated with ibrutinib, Jones et al. report a 14% CR rate (46% ORR).^38^ In similar patients with multiple prior treatments and treated with combined dabrafenib and trametinib, Kreitman et al. report a 78% ORR with a 49% CR rate and a 15% negative MRD rate.^39^

Our data emphasize the need of further real‐life data for HCL patients with two or more relapses. The impact of new and future therapies needs to be assessed in further clinical and observational studies using modern methods of assessment including marrow response and MRD.

## CONFLICT OF INTEREST

The authors have stated explicitly that there are no conflicts of interest in connection with this article.

## ETHICAL STATEMENT

The study was performed in accordance with the Declaration of Helsinki. Patients signed an informed consent at the time of the initial survey.

## AUTHOR CONTRIBUTIONS

All authors had full access to the data in the study and take responsibility for the integrity of the data and the accuracy of the data analysis. *Conceptualization*, J.P. and X.T.; *Methodology*, J.P. and X.T.; *Investigation*, J.P. and X.T.; *Formal Analysis*, J.P. and X.T.; *Resources*, J.P. and X.T.; *Writing‐Original Draft*, J.P. and X.T.; *Writing‐Review & Editing*, J.P. and X.T.; *Supervision*, J.P. and X.T.; *Data Curation*, J.P. and X.T.; *Project Administration*, J.P. and X.T.; *Software*, J.P. and X.T.; *Validation*, J.P. and X.T.

## Data Availability

The data that support the findings of this study are available from the corresponding author upon reasonable request.
